# Heme Oxygenase-1-Mediated Autophagy Protects against Oxidative Damage in Rat Nucleus Pulposus-Derived Mesenchymal Stem Cells

**DOI:** 10.1155/2020/9349762

**Published:** 2020-02-25

**Authors:** Sheng Chen, Sheng Liu, Lei Zhao, Hui Lin, Kaige Ma, Zengwu Shao

**Affiliations:** Department of Orthopaedics, Union Hospital, Tongji Medical College, Huazhong University of Science and Technology, Wuhan 430022, China

## Abstract

Although endogenous nucleus pulposus-derived mesenchymal stem cell- (NPMSC-) based regenerative medicine has provided promising repair strategy for intervertebral disc (IVD) degeneration, the hostile microenvironments in IVD, including oxidative stress, can negatively affect the survival and function of the NPMSCs and severely hinder the endogenous repair process. Therefore, it is of great importance to reveal the mechanisms of the endogenous repair failure caused by the adverse microenvironments in IVD. The aim of this study was to investigate the effect of oxidative stress on the rat NPMSCs and its underlying mechanism. Our results demonstrated that oxidative stress inhibited cell viability, induced apoptosis, and increased the production of reactive oxygen species (ROS) in NPMSCs. In addition, the results showed that the expression level of heme oxygenase-1 (HO-1) increased at an early stage but decreased at a late stage when NPMSCs were exposed to oxidative stress, and the oxidative damages of NPMSCs could be partially reversed by promoting the expression of HO-1. Further mechanistic analysis indicated that the protective effect of HO-1 against oxidative damage in NPMSCs was mediated by the activation of autophagy. Taken together, our study revealed that oxidative stress could inhibit cell viability, induce apoptosis, and increase ROS production in NPMSCs, and HO-1-mediated autophagy might act as a protective response to the oxidative damage. These findings might enhance our understanding on the mechanism of the endogenous repair failure during IVD degeneration and provide novel research direction for the endogenous repair of IVD degeneration.

## 1. Introduction

As a principal cause of disability among the global people, low back pain (LBP) not only brings long-term suffering to individuals but also creates heavy economic burdens for society [1, 2]. Intervertebral disc (IVD) degeneration is considered to be a main contributor to LBP in the modern society [3]. However, the current treatment strategies for IVD degeneration, including physiotherapy, drug therapy, and operation treatment, can only relieve the pain rather than repair the degenerative IVD tissues and reverse the biological functions of IVD [4, 5].

Currently, mesenchymal stem cells- (MSCs-) based biological treatment for IVD degeneration has been studied with increasing interest and showed a promising prospect [6, 7]. We and other researchers from different research groups have isolated, cultured, and identified endogenous IVD MSCs from nucleus pulposus (NP) [8], annulus fibrosus (AF) [9], cartilage endplate (CEP) [10], and stem cell niche areas in IVD [11]. These endogenous MSCs can serve as the seed cells for endogenous repair of IVD degeneration. At the initial stage of the endogenous repair process, the endogenous MSCs are activated and recruited by chemokines, such as stromal cell-derived factor-1*α* [12]. Then, they migrate to degenerative IVD tissues to differentiate into IVD cells to replace the damaged resident cells and (or) protect the damaged cells by some mediators [13]. However, during the process of endogenous repair, IVD-derived MSCs are inevitably exposed to the hostile microenvironments in IVD, including oxidative stress [14], excessive compression [15], acidic pH [16], hypoxia [17], limited nutrition [18], and high osmolarity [19]. These adverse microenvironments can significantly hinder the endogenous repair process by inhibiting the biological behaviors of IVD-derived MSCs and decreasing the number of the cells, and then finally lead to the failure of endogenous repair [13, 20]. Thus, it is urgent to study the effects of the adverse microenvironments on IVD-derived MSCs and related mechanisms, which may contribute to the development of endogenous MSCs-based biological treatment for IVD degeneration.

In recent years, increasing evidence has shown that oxidative stress can do harm to the resident IVD cells and play an essential role in the progression of IVD degeneration [21]. Since IVD cells and IVD-derived MSCs are in the same microenvironments, we speculated that oxidative stress might participate in the failure of endogenous repair for IVD degeneration. Our previous studies demonstrated that excessive compression could inhibit cell viability and increase apoptosis rate of nucleus pulposus-derived mesenchymal stem cells (NPMSCs) by increasing the production of reactive oxygen species (ROS) and inducing oxidative stress, and alleviating oxidative stress partially restored the oxidative damage [15]. Nan et al. [14] also confirmed that hydrogen peroxide, a common inducer of oxidative stress in IVD, could increase ROS production, inhibit cell viability, and increase the apoptosis rate of NPMSCs. However, the specific mechanism of how oxidative stress causes the damage on NPMSCs and how NPMSCs respond to it remains unclear. Therefore, in this study, we aimed to reveal the effect of oxidative stress on the rat NPMSCs and put particular emphasis on its underlying mechanism.

## 2. Materials and Methods

### 2.1. Reagents and Antibodies

Hydrogen peroxide (H_2_O_2_) was purchased from Sangon Biotech (Shanghai, China). Cobalt protoporphyrin IX (CoPP) was purchased from Sigma (USA). Rapamycin (RAP) was purchased from Selleck (USA). The standard MSC expansion medium was purchased from Cyagen Biosciences Inc. (Guangzhou, China). Cell counting kit-8 (CCK-8) was acquired from Dojindo (Japan). ROS detection kit, TUNEL staining, Annexin V-FITC/PI Apoptosis Detection Kit, and Quick Block™ Blocking Buffer were purchased from Beyotime Institute of Biotechnology (Beyotime, China). The rat heme oxygenase-1- (HO-1-) siRNAs were designed and manufactured by Ribobio Co., Ltd. (Guangzhou, China). The mRFP-GFP-LC3 adenovirus was purchased from HanBio Technology Co., Ltd. (Shanghai, China). Primary antibodies against HO-1, *β*-actin, and the secondary antibodies were purchased from Proteintech (Wuhan, China). Primary antibodies against LC3-I/II were purchased from Abcam (UK). Primary antibodies against Beclin-1 were purchased from Cell Signaling Technology (USA).

### 2.2. Isolation and Culture of NPMSCs

All experimental procedures were approved by the Institutional Animal Care and Use Committee of Tongji Medical College of Huazhong University of Science and Technology. The NPMSCs were isolated from male Sprague-Dawley rats (3 months, 250-300 g) with an optimization method as described previously [22]. The obtained NPMSCs were then cultured in the standard MSC expansion medium, which consists of Dulbecco's modified Eagle's medium-low glucose (DMEM-LG), 10% fetal bovine serum (FBS), 2 mmol/L _L_-glutamine, and 1% penicillin-streptomycin. The media were changed twice a week, and the primary cultures were subcultured at a 1 : 2 ratio when cells reached 80–90% confluence. All cell cultures were performed at 37°C with 5% CO_2_. NPMSCs at Passage 3 were used in this study.

### 2.3. Application of H_2_O_2_ and Other Treatments on NPMSC

To study the effect and related mechanisms of oxidative stress on NPMSCs, the cells were treated with H_2_O_2_ for 24 hours at varying doses (0, 40, 80, 120, and 160 *μ*M) or 120 *μ*M H_2_O_2_ for different times (0, 1, 12, and 24 hours). To investigate the role of HO-1 in NPMSCs treated with H_2_O_2_, the cells transfected with negative control siRNA (NC) or HO-1-siRNA were pretreated with 10 *μ*M CoPP for 12 hours and then treated with 120 *μ*M H_2_O_2_ for 1 hour or 24 hours. To determine whether HO-1-mediated autophagy protects against oxidative damage in NPMSCs, the cells transfected with negative control siRNA (NC) or HO-1-siRNA were pretreated with 500 nM RAP for 12 hours and then treated with 120 *μ*M H_2_O_2_ for 1 hour or 24 hours.

### 2.4. CCK-8 Assay

Cell viability was detected using CCK-8. NPMSCs were plated in a 96-well culture plate at a density of 0.5 × 10^4^ per well. After different treatments, the cells were incubated with CCK-8 working solution for 2 hours at 37°C with 5% CO_2_. The absorbance was measured at 450 nm.

### 2.5. ROS Assay

The level of intracellular ROS production was measured by a ROS detection kit. NPMSCs with different treatments were incubated with 2-,7-dichlorofluorescin diacetate (DCFH-DA) at 37°C in the dark for 30 min. After washing twice with PBS, the cells were directly observed under the inverted fluorescence microscope (Olympus, Japan) or measured for the level of intracellular ROS by flow cytometry (BD LSR II, Becton Dickinson).

### 2.6. TUNEL Staining

Cell apoptosis was assayed by TUNEL staining following the manufacturer's protocol. After being fixed in 4% paraformaldehyde for 15 min, the NPMSCs were permeabilized with 0.1% TritonX-100 for 10 min. Then, the cells were washed two times with PBS and incubated with TUNEL staining in the dark at 37°C for 1 h. Apoptotic cells were finally observed under the fluorescence microscope (Olympus, Japan).

### 2.7. Apoptosis Rate

Apoptosis rate of NPMSCs was measured using Annexin V-FITC/PI Apoptosis Detection Kit with the instruction of the manufacturer's protocol. In brief, NPMSCs with different treatments were resuspended in 500 *μ*L binding buffer after trypsinization; then, 5 *μ*L Annexin V-FITC and PI were added. After incubating for 15 min in the dark at room temperature, the apoptosis rate was measured by flow cytometry (BD LSR II, Becton Dickinson).

### 2.8. Western Blotting

NPMSCs were lysed using iced-cold lysis buffer, and then, the total protein was collected by centrifugation (12,000 × g at 4°C for 10 min). After electrophoresis and protein transfer, the PVDF membranes were blocked by Quick Block™ Blocking Buffer and then incubated at 4°C overnight with primary antibodies against HO-1 (1 : 1000), LC3-I/II (1 : 1000), Beclin-1 (1 : 1000), and *β*-actin (1 : 2000). After washing with TBST for three times, the membranes were incubated with secondary antibodies (1 : 5000) for 1 h at room temperature. Then, the proteins were analyzed by the enhanced chemiluminescence method.

### 2.9. Transfection of siRNA

NPMSCs were transfected with NC siRNA and three independent HO-1-siRNAs at a concentration of 50 pmol/10^5^ cell using Lipofectamine™ RNAiMAX Transfection Reagent following the manufacturer's instruction. The target sequences are shown as follows: HO-1-siRNA (#1), GCTAGCCTGGTTCAAGATA; HO-1-siRNA (#2), GGAATTTATGCCATGTAAA; and HO-1-siRNA (#3), CCGTGGCAGTGGGAATTTA. Cells were transfected with siRNAs.

### 2.10. Autophagic Flux Detection

NPMSCs were infected with mRFP-GFP-LC3 adenovirus vector according to the manufacturer's instructions. Then, transfected cells were treated with H_2_O_2_ for different times and observed under a confocal microscope (Nikon, Japan). Autophagic flux was determined by evaluating the number of GFP and mRFP puncta. Free red dots indicated autolysosomes, and yellow dots indicated autophagosomes.

### 2.11. Statistical Analysis

Statistical analysis was performed with GraphPad Prism 6 software (GraphPad Software Inc., USA). All experimental data were obtained from at least three independent experiments and presented as mean ± standard deviation (SD). Unpaired Student's *t*-tests were applied in the analysis of two-group parameters. *P* < 0.05 was considered statistically significant.

## 3. Results

### 3.1. H_2_O_2_-Induced Oxidative Damage on NPMSCs

**Figure 1:**
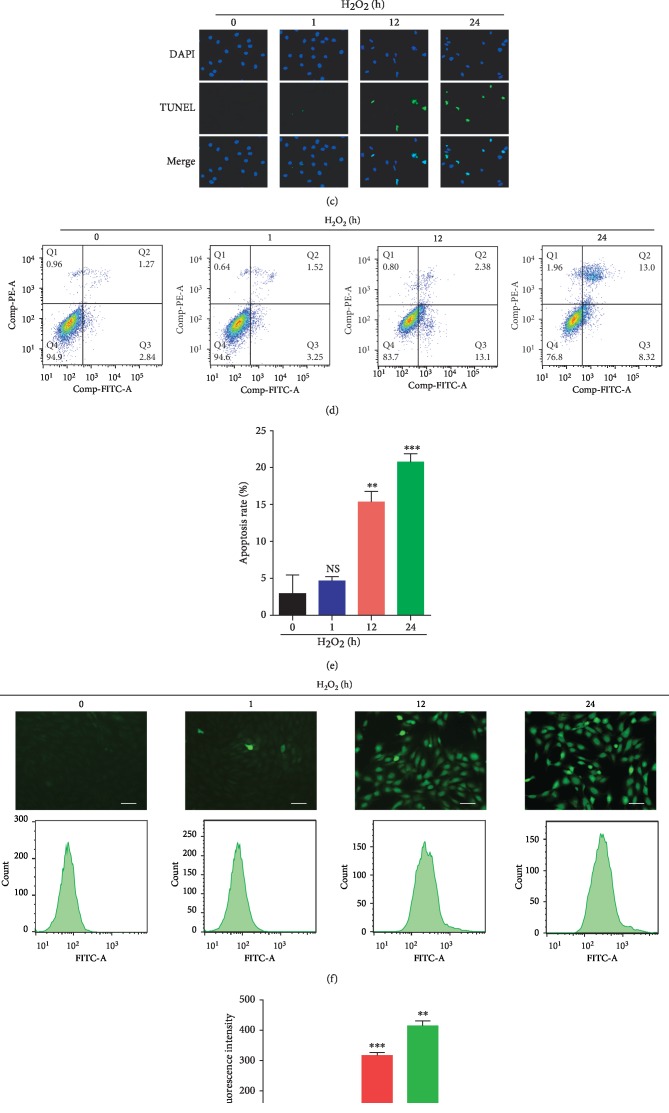
Cell viability, apoptosis, and ROS production of NPMSCs with H_2_O_2_ treatment. (a) Cell viability of NPMSCs treated with different concentrations (0, 40, 80, 120, and 160 *μ*M) of H_2_O_2_ for 24 hours. (b) Cell viability of NPMSCs treated with 120 *μ*M of H_2_O_2_ for different times (0, 1, 12, and 24 hours). (c) TUNEL staining of NPMSCs (bar = 50 *μ*m). (d) Representative images of cell apoptosis detected by flow cytometry after Annexin V/PI dual staining. (e) Summary data showing the apoptosis rate in different groups. The apoptotic cells were stained with Annexin V+/PI- and Annexin V+/PI+. (f) ROS production evaluated by fluorescence staining and flow cytometry (bar = 100 *μ*m). (g) Summary data showing the level of ROS in different groups. NS means no significant difference. The data are expressed as the mean ± SD from three independent experiments (^∗∗^*P* < 0.01, ^∗∗∗^*P* < 0.001 vs. 0 *μ*M of H_2_O_2_).

H_2_O_2_ treatment is a common in vitro model to simulate oxidative stress microenvironment, which has been used in various cell types [23, 24]. In our study, we applied H_2_O_2_ treatment to evaluate the effect of oxidative stress on NPMSCs. CCK8 assay showed that H_2_O_2_ treatment inhibited the cell viability of NPMSCs in a concentration-dependent manner from 0 to 160 *μ*M (Figure 1(a), *P* < 0.01). Considering that the 50% lethal concentration (LC50) was approximately 120 *μ*M, we used this concentration in the following experiments.

As shown in Figure 1(b), short-term treatment of H_2_O_2_ (1 hour) had almost no impact on the cell viability of NPMSCs (*P* > 0.05), while prolonged treatment of H_2_O_2_ (12 and 24 hours) significantly inhibited the cell viability of NPMSCs (*P* < 0.001). To further investigate the influence of oxidative stress on the apoptosis of NPMSCs, TUNEL staining and flow cytometry were used. The results indicated that the apoptosis rate of NPMSCs with short-term treatment of H_2_O_2_ barely changed (*P* > 0.05), but the apoptosis rate of NPMSCs with prolonged treatment of H_2_O_2_ increased obviously (Figures 1(c)–1(e), *P* < 0.01). Oxidative stress always induces the production of ROS, which is closely associated with the decreased cell viability and increased apoptosis. Indeed, our data showed that short-term treatment of H_2_O_2_ did not increase the production of ROS in NPMSCs (*P* > 0.05), while prolonged treatment of H_2_O_2_ significantly increased the production of ROS (Figures 1(f) and 1(g), *P* < 0.01).

### 3.2. H_2_O_2_-Induced Change in Expression Level of HO-1 in NPMSCs

**Figure 2:**
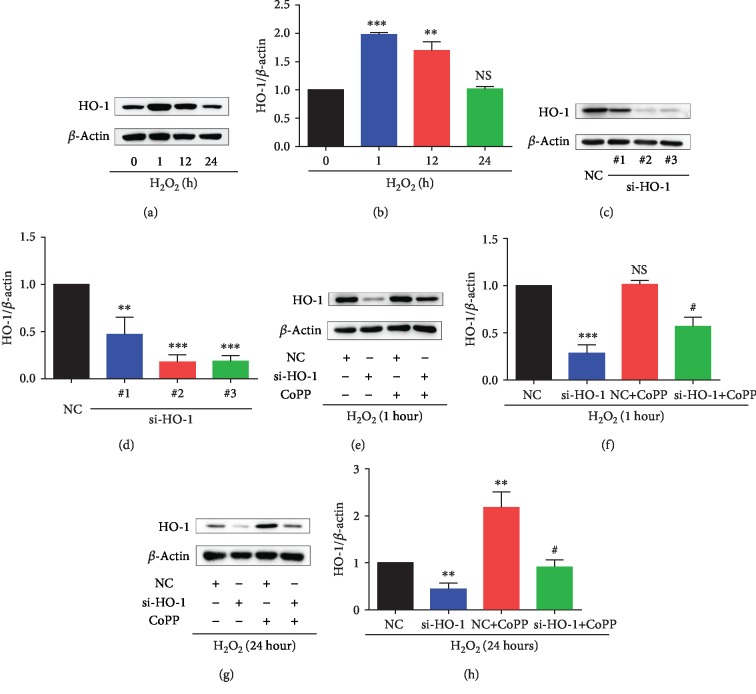
The expression level of HO-1 in NPMSCs with different treatments. (a) The typical western blot bands of HO-1 in NPMSCs treated with H_2_O_2_ for different times. (b) Summary data showing protein levels of HO-1. (c) The typical western blot bands of HO-1 in NPMSCs transfected with negative control siRNA (NC) and three different HO-1-siRNA. (d) Summary data showing protein levels of HO-1. (e) The typical western blot bands of HO-1 in NPMSCs pretreated with negative control siRNA (NC), HO-1-siRNA, or CoPP and then exposed to H_2_O_2_ for 1 hour. (f) Summary data showing protein levels of HO-1. (g) The typical western blot bands of HO-1 in NPMSCs pretreated with negative control siRNA (NC), HO-1-siRNA, or CoPP and then exposed to H_2_O_2_ for 24 hours. (h) Summary data showing protein levels of HO-1. NS means no significant difference. The data are expressed as the mean ± SD from three independent experiments (^∗∗^*P* < 0.01, ^∗∗∗^*P* < 0.001 vs. 0 *μ*M of H_2_O_2_ group, NC group, or NC+H_2_O_2_ treatment group; ^#^*P* < 0.05 vs. si-HO-1+H_2_O_2_ treatment group).

To investigate the role of HO-1in H_2_O_2_-induced oxidative damage in NPMSCs, the expression of HO-1 was detected by western blotting. Interestingly, western blotting analysis demonstrated that the expression level of HO-1 increased significantly at the early stage (1 hour) of treatment with H_2_O_2_ (*P* < 0.001) but then gradually decreased to baseline level (Figures 2(a) and 2(b), *P* > 0.05). To further determine the role of HO-1 in H_2_O_2_-induced oxidative damage, NPMSCs were transfected with NC siRNA and three independent HO-1-siRNAs (#1, #2, and #3). There was no significant effect of transfection on the HO-1 levels in NPMSCs (Supplementary [Supplementary-material supplementary-material-1], *P* > 0.05). The #2 HO-1-siRNA showed the best knocking down effect (*P* < 0.001); we then used the #2 HO-1-siRNA in the remaining experiments of this study (Figures 2(c) and 2(d)). As shown in Figures 2(e)–2(h), HO-1-siRNA obviously downregulated the expression of HO-1 both at the early stage (1 hour) and late stage (24 hours) of treatment with H_2_O_2_, and HO-1 inducer CoPP reversed the expression changes to varying degrees (*P* < 0.05). The results also showed that CoPP significantly upregulated the expression of HO-1 in NPMSCs with prolonged treatment of H_2_O_2_ (Figures 2(g) and 2(h), *P* < 0.01).

### 3.3. HO-1 Protects against Oxidative Damage in NPMSCs

**Figure 3:**
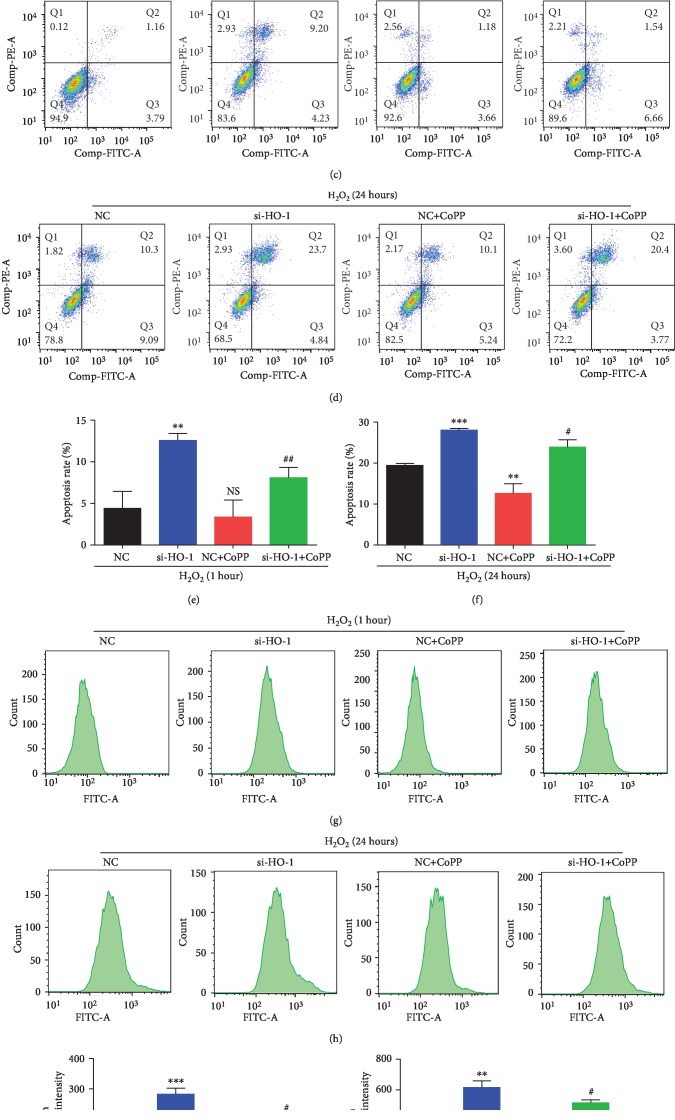
Cell viability, apoptosis, and ROS production of NPMSCs pretreated with negative control siRNA (NC), HO-1-siRNA, or CoPP and then exposed to H_2_O_2_. (a) Cell viability of NPMSCs pretreated with negative control siRNA (NC), HO-1-siRNA, or CoPP and then exposed to H_2_O_2_ for 1 hour. (b) Cell viability of NPMSCs pretreated with negative control siRNA (NC), HO-1-siRNA, or CoPP and then exposed to H_2_O_2_ for 24 hours. (c) Representative images of cell apoptosis detected by flow cytometry. NPMSCs were treated as in (a). (d) Representative images of cell apoptosis detected by flow cytometry. NPMSCs were treated as in (b). (e, f) Summary data showing the apoptosis rate in different groups. The apoptotic cells were stained with Annexin V+/PI- and Annexin V+/PI+. (g) Representative images of ROS production evaluated by flow cytometry. NPMSCs were treated as in (a). (h) Representative images of ROS production evaluated by flow cytometry. NPMSCs were treated as in (b). (i, j) Summary data showing the level of ROS in different groups. NS means no significant difference. The data are expressed as the mean ± SD from three independent experiments (^∗∗^*P* < 0.01, ^∗∗∗^*P* < 0.001 vs. NC+H_2_O_2_ treatment group; ^#^*P* < 0.05 vs. si-HO-1+H_2_O_2_ treatment group).

CCK8 assay revealed that HO-1-siRNA further inhibited the cell viability both at the early stage and late stage of treatment with H_2_O_2_, and upregulating the expression of HO-1 using CoPP partially reversed the decreased cell viability (Figures 3(a) and 3(b), *P* < 0.01). Upregulating the expression of HO-1 using CoPP also increased the cell viability of NPMSCs with prolonged treatment of H_2_O_2_ (Figure 3(b), *P* < 0.01). Flow cytometry showed that HO-1-siRNA further increased the apoptosis rate of NPMSCs both at the early stage and late stage of treatment with H_2_O_2_, and CoPP significantly reversed the increased apoptosis rate (Figures 3(c)–3(f), *P* < 0.05). CoPP also protected against the apoptosis in NPMSCs with prolonged treatment of H_2_O_2_ (Figures 3(d) and 3(f), *P* < 0.01). As expected, our results showed HO-1-siRNA obviously upregulated the ROS level in NPMSCs both at the early stage and late stage of treatment with H_2_O_2_, and CoPP significantly reversed the increased ROS production (Figures 3(g)–3(j), *P* < 0.05). CoPP also inhibited the upregulated level of ROS in NPMSCs with prolonged treatment of H_2_O_2_ (Figures 3(h) and 3(j), *P* < 0.01).

### 3.4. H_2_O_2_-Induced Autophagic Flux Change in NPMSCs

**Figure 4:**
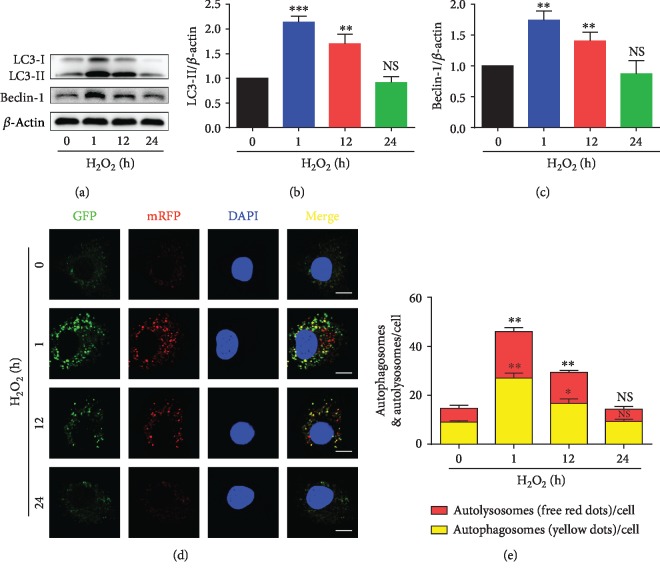
The autophagic flux in NPMSCs with H_2_O_2_ treatment. (a) The typical western blot bands of LC3 and Beclin-1 in NPMSCs treated with H_2_O_2_ for different times. (b, c) Summary data showing protein levels of LC3 II and Beclin-1. (d) Representative fluorescence images showing the autophagosomes (yellow dots) and autolysosomes (free red dots) (bar = 5 *μ*m). (e) Summary data showing the number of autophagosomes and autolysosomes. NS means no significant difference. The data are expressed as the mean ± SD from three independent experiments (^∗^*P* < 0.05, ^∗∗^*P* < 0.01, and ^∗∗∗^*P* < 0.001 vs. 0 *μ*M of H_2_O_2_ group).

In order to monitor the change of autophagic flux over the time of H_2_O_2_ treatment, the expressions of LC3 II and Beclin-1 were detected by western blotting, and the number of autolysosomes as well as autophagosomes was evaluated using mRFP-GFP-LC3 adenovirus vector. As shown in Figures 4(a)–4(c), the expressions of LC3 II and Beclin-1 increased significantly at the early stage of treatment with H_2_O_2_ (*P* < 0.01) but then gradually decreased to baseline level (*P* > 0.05). Predictably, fluorescence staining showed that the number of autolysosomes and autophagosomes remarkably increased at the early stage of treatment with H_2_O_2_ (*P* < 0.05) but barely changed at the late stage (Figures 4(d) and 4(e)). These results suggested that autophagy was activated and might play a key role in protecting against oxidative damage at the early stage of treatment with H_2_O_2_.

### 3.5. HO-1 Regulated Autophagy Activation in NPMSCs with H_2_O_2_ Treatment

**Figure 5:**
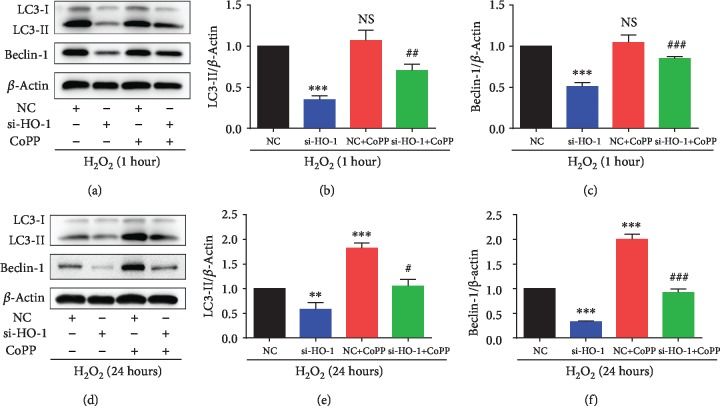
HO-1 regulated the expressions of autophagy-related proteins in NPMSCs with different treatments. (a) The typical western blot bands of LC3 and Beclin-1 in NPMSCs pretreated with negative control siRNA (NC), HO-1-siRNA, or CoPPand then exposed to H_2_O_2_ for 1 hour. (b, c) Summary data showing protein levels of LC3 II and Beclin-1. (d) The typical western blot bands of LC3 and Beclin-1 in NPMSCs pretreated with negative control siRNA (NC), HO-1-siRNA, or CoPP and then exposed to H_2_O_2_ for 24 hours. (e, f) Ssummary data showing protein levels of LC3 II and Beclin-1. NS means no significant difference. The data are expressed as the mean ± SD from three independent experiments (^∗∗^*P* < 0.01, ^∗∗∗^*P* < 0.001 vs. NC+H_2_O_2_ treatment group; ^#^*P* < 0.05, ^##^*P* < 0.01, and ^###^*P* < 0.001 vs. si-HO-1+H_2_O_2_ treatment group).

Then, we investigated whether HO-1 regulated the autophagic flux in NPMSCs with H_2_O_2_ treatment. Western blotting analysis revealed that HO-1-siRNA inhibited short-term H_2_O_2_ treatment-induced autophagy activation by downregulating the expression levels of LC3 II and Beclin-1 in NPMSCs, and CoPP could partially reverse it (Figures 5(a)–5(c), *P* < 0.01). Similarly, HO-1-siRNA downregulated the expressions of LC3 II and Beclin-1 in NPMSCs with prolonged H_2_O_2_ treatment, and CoPP partially reversed the change (Figures 5(d)–5(f), *P* < 0.05). Moreover, CoPP upregulated the expression levels of LC3 II and Beclin-1 in NPMSCs with prolonged H_2_O_2_ treatment (Figures 5(d)–5(f), *P* < 0.001). The results indicated that HO-1 mediated the activation of autophagy in NPMSCs with H_2_O_2_ treatment.

### 3.6. HO-1-Meadiated Autophagy Protects against Oxidative Damage in NPMSCs

**Figure 6:**
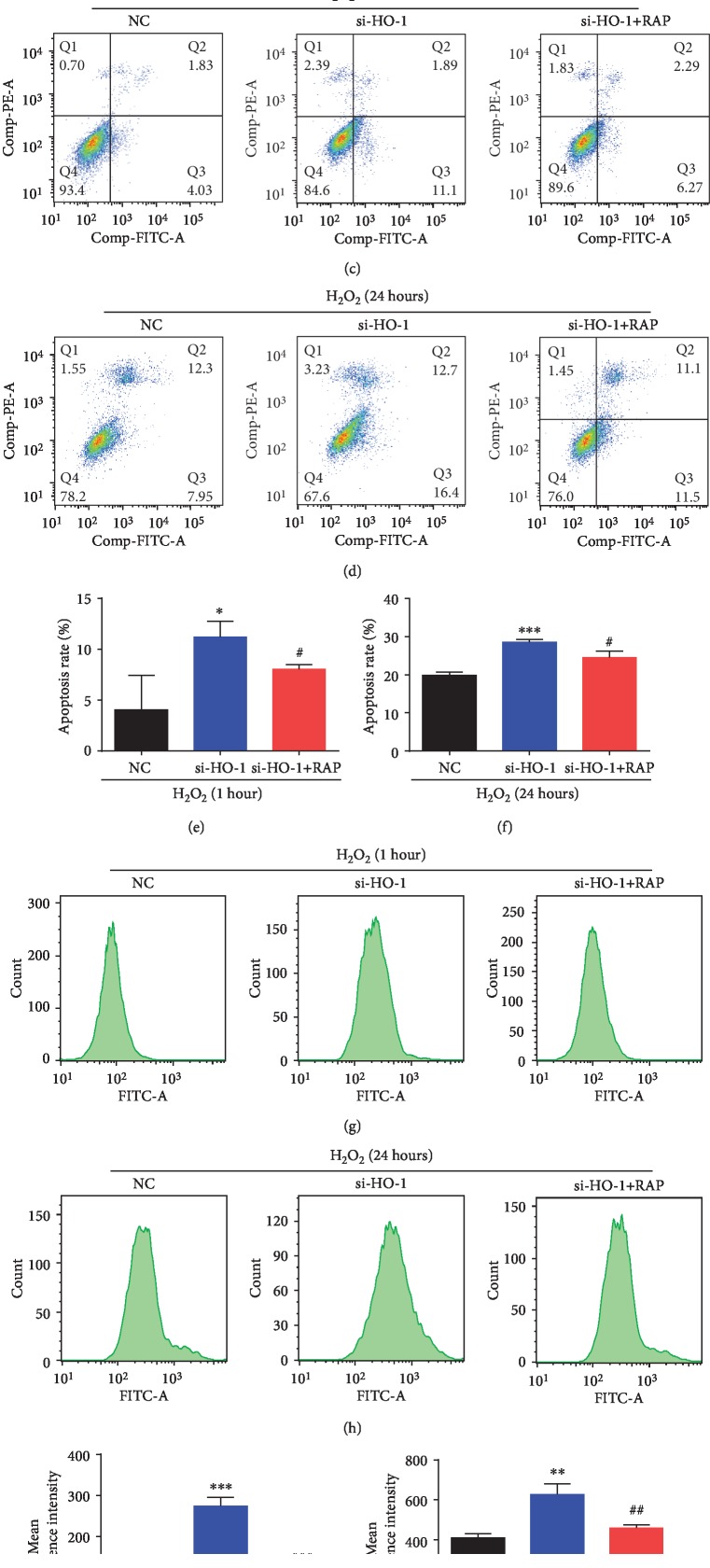
Cell viability, apoptosis, and ROS production of NPMSCs pretreated with negative control siRNA (NC), HO-1-siRNA, or RAP and then exposed to H_2_O_2_. (a) Cell viability of NPMSCs pretreated with negative control siRNA (NC), HO-1-siRNA, or RAP and then exposed to H_2_O_2_ for 1 hour. (b) Cell viability of NPMSCs pretreated with negative control siRNA (NC), HO-1-siRNA, or RAP and then exposed to H_2_O_2_ for 24 hours. (c) Representative images of cell apoptosis detected by flow cytometry. NPMSCs were treated as in (a). (d) Representative images of cell apoptosis detected by flow cytometry. NPMSCs were treated as in (b). (e, f) Summary data showing the apoptosis rate in different groups. The apoptotic cells were stained with Annexin V+/PI- and Annexin V+/PI+. (g) Representative images of ROS production evaluated by flow cytometry. NPMSCs were treated as in (a). (h) Representative images of ROS production evaluated by flow cytometry. NPMSCs were treated as in (b). (i, j) Summary data showing the level of ROS in different groups. The data are expressed as the mean ± SD from three independent experiments (^∗^*P* < 0.05, ^∗∗^*P* < 0.01, and ^∗∗∗^*P* < 0.001 vs. NC+H_2_O_2_ treatment group; ^#^*P* < 0.05, ^##^*P* < 0.01, and ^###^*P* < 0.001 vs. si-HO-1+H_2_O_2_ treatment group).

To investigate whether HO-1-mediated autophagy was involved in the protection of NPMSCs from oxidative damage, the autophagy activator rapamycin (RAP) was used. The results demonstrated that RAP restored the HO-1-siRNA-induced decrease of cell viability (Figures 6(a) and 6(b), *P* < 0.01), increase of apoptosis rate (Figures 6(c)–6(f), *P* < 0.05), and upregulation of ROS level (Figures 6(g)–6(j), *P* < 0.01) both at the early stage and late stage of treatment with H_2_O_2_. The results above suggested that HO-1 protected against oxidative damage in NPMSCs, at least in part, by activating autophagy.

## 4. Discussion

Recently, increasing studies have indicated that oxidative stress plays a critical role in the initiation and development of IVD degeneration [25, 26]. In the process of oxidative stress, excess amounts of ROS, including H_2_O_2_, hydroxyl radicals, superoxide anions, and hypochlorite ions are generated in IVD, which can cause the disruption of extracellular matrix (ECM) homeostasis, inflammatory response, and cell loss of IVD cells, such as NP cells, directly resulting in IVD degeneration [27]. In consideration of the fact that NPMSCs and NP cells are in the same oxidative stress microenvironment, we speculated that oxidative stress could probably lead to IVD degeneration indirectly by impairing NPMSCs and hindering endogenous repair.

To test the hypothesis, we treated NPMSCs with H_2_O_2_ for different times in this study. Our data showed that short-term treatment of H_2_O_2_ had almost no impact on the cell viability, apoptosis rate, and ROS production in NPMSCs, while prolonged treatment of H_2_O_2_ significantly inhibited the cell viability, increased the apoptosis rate, and elevated the level of ROS. The results suggested that NPMSCs might initiate a self-protection mechanism to respond to oxidative damage at the early stage of H_2_O_2_ treatment but failed at the late stage, which had been observed in many cell types. Fan et al. [28] found that short-term oxidative stress could rapidly facilitate mitophagy to reduce oxidative damage in bone marrow-derived MSCs, and prolonged oxidative stress inhibited mitophagy and enhanced apoptosis. In endplate chondrocytes, results from Chen et al. [29] also demonstrated that autophagy could be activated at the early stage of oxidative stress to protect against apoptosis, but prolonged oxidative stress decreased the autophagic flux. However, the self-adaptive protection mechanism of NPMSCs treated with H_2_O_2_ remains unclear. Therefore, we then tried to reveal the self-adaptive protection mechanism, which might provide a novel target to reverse endogenous repair failure and promote the development of therapies for IVD degeneration.

HO-1, which has been identified in many pathophysiological scenarios, serves a critical biological function as the rate-limiting enzyme in the metabolism of heme to generate biliverdin, carbon monoxide, and iron [30, 31]. As a cellular stress protein, HO-1 can function as one of the most important factors of cell adaptation to oxidative stress and exert cytoprotective effects against environment stress-induced inflammation and cell death [32]. Recent evidence revealed that HO-1 could alleviate IVD degeneration by attenuating inflammation-induced apoptosis and imbalance between ECM anabolism and catabolism in NP cells [33, 34]. Then, we focused on HO-1 and investigated whether HO-1 was involved in the self-adaptive protection mechanism of NPMSCs treated with H_2_O_2_. Our results showed that the expression level of HO-1 increased significantly at the early stage of treatment with H_2_O_2_ but then gradually decreased to baseline level. The expression change of HO-1 implied that HO-1 might be upregulated at the early stage of H_2_O_2_ treatment to protect against the oxidative damage but failed at the late stage due to the decreased expression. To verify this, HO-1-siRNA and HO-1 inducer CoPP were applied. The results demonstrated that HO-1-siRNA further inhibited cell viability, increased the apoptosis rate, and elevated the ROS level of NPMSCs both at the early stage and late stage of treatment with H_2_O_2_, and CoPP significantly reversed these changes. Moreover, CoPP could restore the prolonged treatment of H_2_O_2_-induced decrease of cell viability, increase of apoptosis, and ROS production in NPMSCs. All the results suggested that HO-1 played a key role in self-adaptive protection against oxidative damage in NPMSCs.

Autophagy is a highly conserved cellular recycling process, which involves self-degradation and reconstruction of misfolded proteins and damaged organelles, and acts as a main cytoprotective system to maintain organellar quality control and nutrient homeostasis [35–37]. Previous studies reported that there was a link between the activity of HO-1 and the activation of autophagy [38–40]. In line with previous studies, we observed that the autophagic flux followed the same changing trend as the expression of HO-1 in NPMSCs with H_2_O_2_ treatment for different times. Furthermore, HO-1-siRNA significantly decreased the autophagic flux both at the early stage and at the late stage of treatment with H_2_O_2_, and CoPP obviously restored the changes. The data suggested that HO-1 could mediate the activation of autophagy in NPMSCs with H_2_O_2_ treatment. But it was still unclear whether HO-1-mediated autophagy was involved in self-adaptive protection against oxidative damage in NPMSCs. To find out, we then used autophagy activator rapamycin. The results showed that rapamycin could partially reverse HO-1 knockdown-induced increased cell damage both at the early stage and late stage of treatment with H_2_O_2_. Of note, the anti-apoptosis effect of rapamycin on NPMSCs was not obvious. It indicated that HO-1-mediated autophagy might not be the sole protective mechanism against oxidative stress in NPMSCs. Thus, further studies to explore the specific molecular mechanism are needed.

In summary, our results in the present study demonstrated that oxidative stress could impair NPMSCs by inhibiting cell viability, increasing apoptosis, and elevating ROS level. And HO-1-mediated autophagy was involved in the self-adaptive protection against oxidative damage. Our findings might enhance our understanding on the mechanism of IVD degeneration and provide a new target to reverse endogenous repair failure and then restore degenerative IVDs.

## Data Availability

The data used to support the findings of this study are available from the corresponding author upon request.

## Supplementary Materials

Supplementary Materials Supplementary Figure 1: there was no significant effect of transfection on the HO-1 levels in NPMSCs. (a) The typical western blot bands of HO-1 in NPMSCs treated with or without negative control siRNA (NC). (b) Summary data showing protein levels of HO-1. NS means no significant difference. The data are expressed as the mean ± SD from three independent experiments.
