# Supplementary material for: Heme Oxygenase-1-Mediated Autophagy Protects against Oxidative Damage in Rat Nucleus Pulposus-Derived Mesenchymal Stem Cells
Source: Oxid Med Cell Longev. 2020 Feb 25;2020:9349762. doi: 10.1155/2020/9349762 (PMC7063211; doi:10.1155/2020/9349762)
Supplement: Supplementary Materials — Supplementary Figure 1: there was no significant effect of transfection on the HO-1 levels in NPMSCs. (a) The typical western blot bands of HO-1 in NPMSCs treated with or without negative control siRNA (NC). (b) Summary data showing protein levels of HO-1. NS means no significant difference. The data are expressed as the mean ± SD from three independent experiments. [file 9349762.f1.docx]

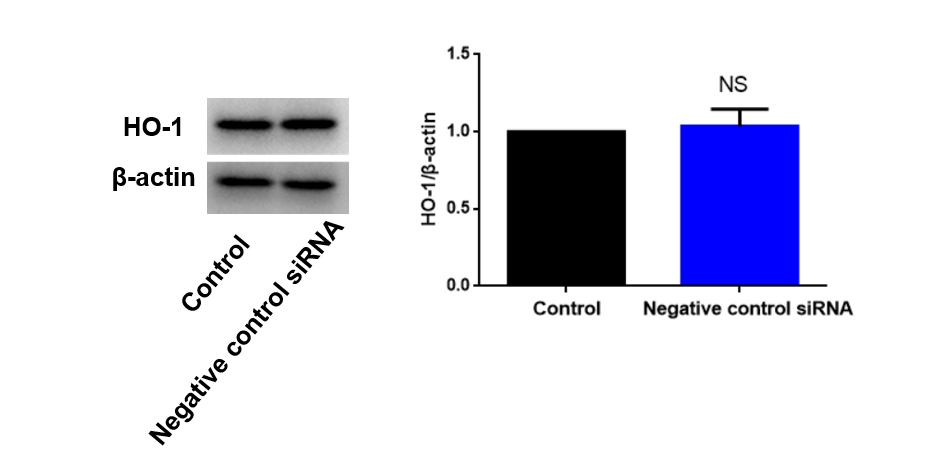


1. **(b)**

**Supplementary Figure 1:** There was no significant effect of transfection on the HO-1 levels in NPMSCs. **(a)** The typical western blot bands of HO-1 in NPMSCs treated with or without negative control siRNA (NC). **(b)** Summary data showing protein levels of HO-1. NS means no significant difference. The data are expressed as mean ± SD from three independent experiments.
